# iPS Cells for Modelling and Treatment of Retinal Diseases

**DOI:** 10.3390/jcm3041511

**Published:** 2014-12-19

**Authors:** Fred K. Chen, Samuel McLenachan, Michael Edel, Lyndon Da Cruz, Peter J. Coffey, David A. Mackey

**Affiliations:** 1 Centre for Ophthalmology and Visual Science (Incorporating Lions Eye Institute), The University of Western Australia, Perth WA 6009, Australia; E-Mails: SamuelMcLenachan@lei.org.au (S.M.); davidmackey@lei.org.au (D.A.M.); 2 Ophthalmology Department, Royal Perth Hospital, Perth WA 6009, Australia; 3 Control of Pluripotency Laboratory, Department of Physiological Sciences I, Faculty of Medicine, University of Barcelona, Hospital Clinic, Casanova 143, Barcelona 08036, Spain; E-Mail: edel.michael@gmail.com; 4 School of Medicine and Pharmacology, University of Western Australia, Crawley, Perth WA 6009, Australia; 5 Faculty of Medicine, Westmead Children’s Hospital, Division of Paediatrics and Child Health, University of Sydney Medical School, Sydney NSW 2145, Australia; 6 Developmental and Stem Cell Biology, Victor Chang Cardiac Research Institute, Sydney NSW 2145, Australia; 7 Department of Vitreoretinal Surgery, Moorfields Eye Hospital, London EC1V 2PD, UK; E-Mail: lyndon.dacruz@moorfields.nhs.uk; 8 Division of Cellular Therapy, Institute of Ophthalmology, University College of London, London EC1V 9EL, UK; E-Mail: p.coffey@ucl.ac.uk

**Keywords:** age-related macular degeneration, inherited retinal disease, retinal dystrophy, macular dystrophy, retina, induced pluripotent, stem cells, cell transplantation, disease modelling, genetic diagnosis

## Abstract

For many decades, we have relied on immortalised retinal cell lines, histology of enucleated human eyes, animal models, clinical observation, genetic studies and human clinical trials to learn more about the pathogenesis of retinal diseases and explore treatment options. The recent availability of patient-specific induced pluripotent stem cells (iPSC) for deriving retinal lineages has added a powerful alternative tool for discovering new disease-causing mutations, studying genotype-phenotype relationships, performing therapeutics-toxicity screening and developing personalised cell therapy. This review article provides a clinical perspective on the current and potential benefits of iPSC for managing the most common blinding diseases of the eye: inherited retinal diseases and age-related macular degeneration.

## 1. Introduction

The ability to convert a differentiated somatic cell from a patient into a pluripotent stem cell has provided new tools for studying organ development and genotype-phenotype relationships. Three-dimensional tissue structures and cells derived from these induced pluripotent stem cells (iPSCs) are now being used to screen and test the therapeutic and toxic effects of potential pharmacologic agents and gene therapies. More importantly, iPSCs could also be used to provide an easily accessible source of tissue for autologous cellular therapy. To date, the greatest potential benefit of iPSC technology is in the treatment of retinal diseases.

**Figure 1:**
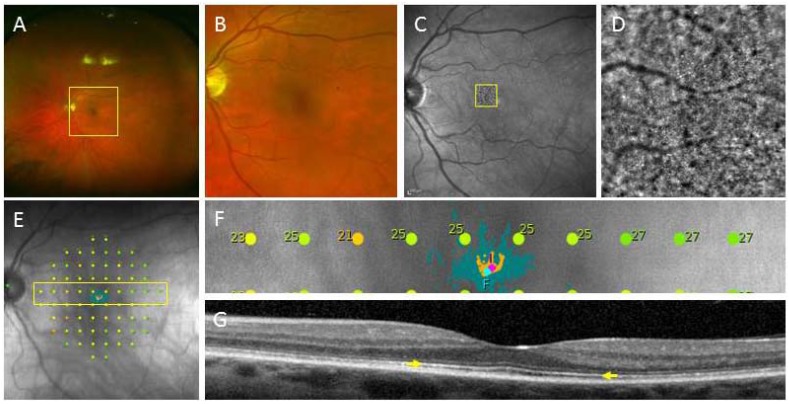
An example of high-resolution retinal images from a patient with hydroxychloroquine toxicity. (**A**) Wide-field colour photography; (**B**) Zoomed-in colour image highlighted by the yellow box in (**A**) of the macular region showing no obvious abnormality; (**C**) Near-infrared reflectance image of the macula showing no obvious abnormality; (**D**) Adaptive optics retinal image highlighted by the yellow box in (**C**) showing the loss of wave-guiding cone outer segments in the perifoveal region; (**E**) Microperimetry showing reduced sensitivity to light in the macular region; (**F**) Zoomed-in image of the perifoveal region showing reduced sensitivity (<25 dB is abnormal); (**G**) Corresponding optical coherence tomography through the fovea showing no obvious loss of the ellipsoid zone of the photoreceptors (yellow arrow).

The retina is a complex neurovascular tissue within the eye. It contains a network of neurons nourished by the retinal and choroidal circulations. Specialised neuronal cells, called rod and cone photoreceptors, capture light that enters into the eye. Through phototransduction within the photoreceptors and downstream neural processing by the bipolar, amacrine, horizontal and ganglion cells within the retina, light signals are transmitted to the primary and secondary visual cortex of the brain to enable visual sensation. The functions of these specialised neuronal cells are supported by the Muller glial cells and the retinal pigment epithelium (RPE). The ease of visualising retinal neurons and assessing the structure-function correlation in detail using readily available imaging devices will facilitate the *in vivo* clinical translation of iPSC technology in the diagnosis and treatment of retinal diseases (Figure 1).

**Figure 2:**
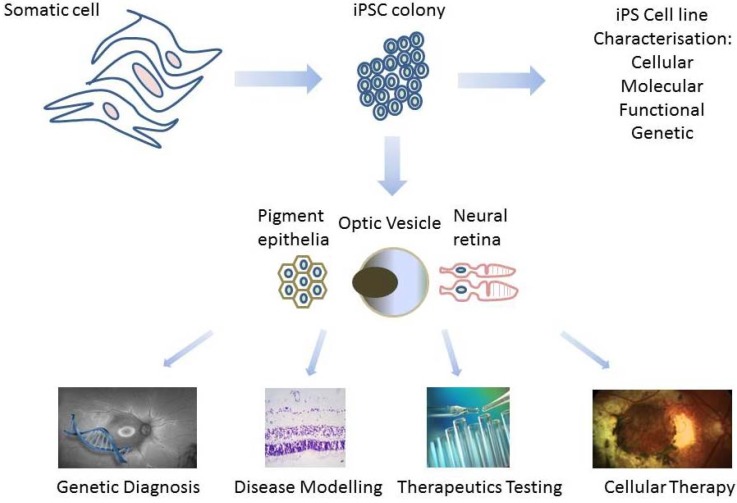
A somatic cell from the patient is used to derive induced pluripotent stem cells (iPSCs). The iPSC colonies are characterised to ensure pluripotency markers are present, they form teratoma or embryoid body and they have stable chromosomes. It may take up to three months to derive and validate iPSC lines. The validated iPSC colonies are differentiated to form optic vesicle structures, which contain retinal pigment epithelium and neural retinal cells. Mature retinal cells can be used for confirming the pathogenicity of newly-discovered genetic variants, modelling of developmental or degenerative retinal disease, testing of pharmacologic agents or gene therapy and autologous cellular therapy.

Among hundreds of human retinal diseases, the most significant are age-related macular degeneration (AMD) and the inherited retinal diseases (IRDs). Both AMD and IRDs are neither preventable nor curable, and they remain the most significant causes of irreversible blindness. The underlying processes leading to retinal cell death range from cell-autonomous mechanisms related to single gene mutations to complex gene-metabolic-environment interaction, resulting in extracellular remodelling, abnormal angiogenesis, chronic inflammation, defective lipid metabolism and oxidative injury, as proposed in AMD [1]. The discovery of the pathological basis of these diseases was made possible through clinical observation using detailed retinal imaging techniques, human genetic studies, histology of post-mortem, enucleated or aborted foetal eyes, immortalised cell line culture systems and animal models of retinal diseases. However, in routine clinical practice, retinal diagnosis is rarely based on retinal histology because of the significant morbidity associated with retinal biopsy and the ease in making a diagnosis, because the retina is easily visualised. The availability of iPSC technology provides an opportunity to obtain retinal tissue without retinal biopsy. There are now several examples in which iPSC-derived retinal cells are used to confirm the clinical and genetic diagnosis of IRDs [2,3], understand the molecular mechanisms of developmental anomalies of the eye [4] and explore the cellular mechanisms of specific genetic mutations [5,6,7,8]. In addition to improving diagnostic capability, the use of iPSCs in clinical practice could also lead to new treatments for retinal diseases (Figure 2).

Central to most blinding retinal diseases is the loss of cone photoreceptors. Strategies to preserve or replace cone cells are under intense investigation. Cones can be preserved by: (1) anti-oxidant therapy; (2) pharmacological therapy that provides neuroprotection; (3) gene correction therapy; and (4) cell-based therapy to provide support to cone cells (e.g., RPE or rod cell transplantation). Lost cone cells can be replaced by: (1) transplantation of patient-specific or allogeneic photoreceptor precursors (along with supporting cells); (2) recruitment of endogenous cells to differentiate into new photoreceptor or to become light-responsive cells (optogenetics); or (3) implantation of *epi*-retinal, *sub*-retinal, suprachoroidal or optic nerve visual prostheses [9,10,11]. Some of these treatment modalities have been investigated in cell culture systems and animal models, and many of these have also been tested in phase I/II clinical trials [12,13,14,15]. A major limitation of clinical therapeutics trial in IRDs is the vast heterogeneity of the underlying genetic mutation. Many of the approaches to preserve cones may only be suitable for one genetic variant, but not another, despite a similar clinical phenotype. Given the rarity of many IRDs, randomised clinical trials are not feasible. As an alternative, iPSC-derived retinal tissue from many patients with IRDs can now be tested *in vitro*, simultaneously, in a pre-clinical study, for the potential dose-therapeutic effect response and toxicity of various pharmacologic agents or gene therapies. As genomic editing techniques are emerging and iPSCs are being used as a cell source for replacing lost retinal cells, we now also have the capability of eliminating specific mutations prior to retinal differentiation, thus providing the option of autologous transplantation even to patients with IRDs [16].

There are several excellent reviews on the use of human iPSCs in the study of retinogenesis, modelling retinal disease, screening of therapeutics and cell replacement therapy in both AMD and IRDs [17,18,19,20,21,22]. The purpose of this review is to provide an update, from a clinical perspective, on the potential for using iPSC technology in routine clinical care of patients with retinal diseases. It will expand on clinically relevant issues related to laboratory techniques to derive clinical grade iPSC-retina and illustrate examples in which iPSC technology has translated into patient care.

## 2. Derivation of Patient-Specific Retinal Cells from iPSC for Clinical Use

The availability of human retinal tissue and pure populations of specific types of retinal cells is critical to our ability to diagnose and treat retinal diseases. Allogeneic sources of retinal tissue and cells can be obtained from donor eyes or cell lines. However, these are not clinically useful for confirming genetic diagnosis of a patient or for autologous cellular therapy. Access to patient-specific retinal tissue requires an intraocular procedure, called a vitrectomy, followed by detachment of the retina, retinectomy, laser retinopexy and a vitreous substitute to provide a temporary tamponade. Although this type of procedure is rarely performed for obtaining retinal tissue for the diagnosis of vitreoretinal lymphoma, there are significant blinding complications, such as retinal detachment, and the harvested retinal tissue will not be of adequate quantity or quality for disease modelling, retinal regenerative therapy or screening new therapeutics. Therefore, there is a clinical need for obtaining patient-specific retinal cells without the need to perform retinal biopsy.

### 2.1. Creating iPSC from Patients

#### 2.1.1. Using Pluripotent Stem Cells

An alternative method to obtain patient-specific retinal cells is to use patient-derived adult stem cells for differentiation into retinal lineages. Retinal neural and pigment epithelial progenitor cells [23,24] have been found in the adult retina, but access to these cells is also limited, as they will require vitrectomy surgery, making them equally unsuitable for clinical use in testing therapeutics and administering personalised cell therapy. Multipotent neural stem cells capable of generating retinal lineages have also been found in the ciliary margin zone and corneoscleral limbus [25,26,27,28]. The former source is located adjacent to the lens within the eye, and it is even more difficult to access than the retina. In contrast, limbal tissue is routinely harvested by corneal surgeons for autologous limbal transplantation. Despite the ease of limbal cell harvesting and the long-term safety of the limbal graft donor site [29,30], its use for retinal regeneration and disease modelling has not yet been explored due to limited data on the ability for *in vitro* expansion and the potential for differentiation into all retinal cell types.

Unlike adult stem cells that are multipotent or unipotent, *i.e.*, committed to specific cell types, pluripotent stem cells (PSCs) have a capacity for unlimited self-renewal (hence, large quantities of cells) and differentiation into any somatic cell type, including all classes of retinal cells. One source of PSC is the embryonic stem cells (ESCs), harvested from the inner cell mass of the blastocyst, from which each of the three germ layers—the endoderm, ectoderm and mesoderm—can be derived. However, ESCs are derived from discarded surplus embryos, and this is not patient-specific. Human ESC-derived RPE is currently being used in several clinical trials, but recipients are being immunosuppressed, because of the potential risk of graft rejection [12].

More recently, PSCs can also be generated by dedifferentiating a terminally differentiated patient-specific adult somatic cell, such as a fibroblast, into a pluripotent state by nuclear reprogramming. There are three established methods to induce pluripotency: (1) transfer of the nucleus of a differentiated cell into an enucleated oocyte (nucleus removed), so that pluripotency genes within the somatic cell genome are activated by the regulators within the oocyte cytoplasm (nuclear transfer) [31]; (2) fusion of a somatic cell with an ESC to create a hybrid or heterokaryon in which pluripotency regulators override cell differentiation regulators (cell fusion) [32]; and (3) induced overexpression of specific pluripotency transcriptional factors through transfection of an adult somatic cell with integrating virus, non-integrating virus, plasmids, mRNA or even exposure to protein or small molecules (induced pluripotent stem cell (iPSC) reprogramming) [33].

#### 2.1.2. Induced Pluripotent Stem Cells

Since the original description of the iPSC protocol by Yamanaka [34], there has been significant development in the reprogramming approach, and many types of somatic cells have been successfully induced into a pluripotent state. Conceptually, this is a two-step process of (1) nuclear reprogramming of a chosen somatic cell into several clones of iPSC and (2) validation of the pluripotency of the various clones of iPSC to select the most suitable clone for the specific purpose that iPSCs will be used, for diagnostics or therapeutics. The time lag from biopsy to obtain patient’s somatic cells to full validation of the best iPSC clone may take 2–3 months. Several factors will influence the choice of somatic cell for deriving iPSC.

For therapeutic purposes, such as autologous cell replacement therapy, the ideal iPSC clone should be derived from an easily accessible somatic cell type in facilities that comply with good manufacturing practice guidelines related to cell therapy. Both adult stem cells and differentiated cells have been used to derive good quality iPSC lines. Although adult stem cells may already express some of the pluripotency-related genes, their expression is significantly lower than that seen in ESC or iPSC. Therefore, the same protocol for deriving iPSC is generally required for adult stem cells as for differentiated cells. Cells that proliferate well also reprogram well. However, there is significant variability in genetic and epigenetic patterns and the degree of reprogramming, even between iPSC clones from the same cell source. Hu *et al.* showed that iPSCs derived from RPE retain a “memory” of cellular origin with respect to the propensity for differentiation back to RPE [35]. However, it will not be feasible to use patients’ RPE as a source for deriving iPSC, due to surgical complications associated with tissue harvest. Furthermore, even without “memory” in source cells, RPE and neuroretinal cells have been generated readily from iPSC derived from cells of diverse background, such as cord blood cell, lymphocyte, keratinocyte, adipocyte and fibroblast [2,4,36,37,38]. Another easily accessible source of somatic cells is the ocular surface. The potential to generate iPSC from cells on the ocular surface (corneal epithelium and limbal niche) warrants further investigation, as they can potentially be reprogrammed to pluripotency without the introduction of transcriptional factors, as shown in rodent limbal-derived neurospheres [39,40]. In contrast to autologous transplantation of iPSC-derived retinal cells, special consideration needs to be given to the ease of transport and storage of somatic cells for deriving iPSC for the purpose of genetic diagnosis, disease modelling and high throughput drug screening. In this situation, blood-derived cells (activated T lymphocytes and endothelial progenitor cells) may be preferable, as they are easily collected, transported, isolated and stored [41,42,43].

**Figure 3:**
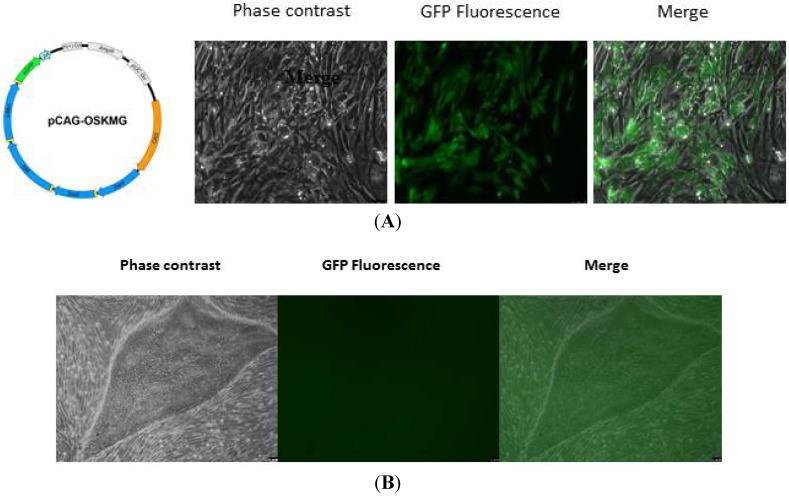
Retrovirus vector for induced pluripotent stem cell (iPSC) reprogramming. (**A**) Map of polycistronic retroviral vector. Human fibroblasts two days after infection with polycistronic GFP Oct4/Sox2/Klf4/cMyc; (**B**) iPSC after four weeks post infection negative for GFP indicating that the transgene is silenced in iPSC clone.

The reprogramming protocol should preferably avoid the use of viruses, such as retroviruses, that were used to create the first human iPSC [34,44]. Non-integrating viral vectors, DNA plasmids, modified RNA, protein and small molecules have all been reported to induce a pluripotent state in a somatic cell [45,46,47,48,49,50]. There is no one perfect methodology for creating iPSC for all types of clinical use. The main trade-off for the potential mutagenesis by integrating virus is the lower efficiency and higher cost associated with non-integrating methods. There have also been variations on the transcriptional factors used for reprogramming since the original description by Yamanaka (OCT4, SOX2, KLF4 and c-MYC) and Thomson (OCT4, SOX2, NANOG and LIN28) (Figure 3) [34,44]. Some protocols also use additional small molecules, such as 5-aza-deoxycytidine, valproic acid or ascorbic acid, to modify the epigenetic environment and enhance the efficiency and accuracy of nuclear reprogramming. Ultimately, these protocol modifications will also have an impact on the cost and quality of the human iPSC line and the suitability for clinical application, such as autologous transplantation. Regardless of the cell source and reprogramming protocol, successful generation of retinal tissue from iPSCs will depend on the skills of the operator in identifying the “right” iPSC clones for retinal differentiation.

#### 2.1.3. Validation of Human iPSC Lines

The key defining features of iPSC are the self-renewal capacity and the ability to produce all three germ layers. Not all iPSC clones generated from the same somatic cell line from the same patient will be fully reprogrammed or truly pluripotent, and the efficiency of various protocols in generating iPSC clones can vary from 1:100 to 1:10,000. Screening to distinguish partially and fully reprogrammed colonies may add further delay and cost to the generation of patient-specific iPSC lines, and the thoroughness of this process depends on the clinical reasons for deriving the iPSC. It has been suggested that between five and 10 clones may need to be isolated for characterisation and future differentiation, because not all clones will have the same propensity for retinal lineage derivation, despite their potential [6,7,51,52].

Four techniques are used for characterising and subsequently selecting iPSC clones: cellular, molecular, functional and genetic (Table 1). The extent of characterisation required will again depend on the purpose of generating iPSC. Less rigorous criteria may be sufficient for genetic diagnosis and disease modelling compared to drug screening and cellular therapy. However, a minimum set of criteria for establishing putative iPSC has been recommended by the European Consortium of stem cell research (the ESTOOLS project).

The unpredictable variability between clones may be related to the somatic origin of iPSC, the reprogramming technique or the intrinsic clonal variability within the individual. Furthermore, equal performance of iPSC clones against the same “pluripotency” tests does not translate to equal propensity for retinal lineage derivation. Further investigation is required to establish a selection screen and criteria for reducing clonal variation and identifying iPSC clones that have optimal retinal differentiation propensity. It can be envisaged that different criteria for establishing pluripotency may emerge for diagnostic and therapeutic use of iPSC.

### 2.2. Creating Retinal Tissue from iPSC

#### 2.2.1. Derivation of Retina Lineages

The fundamental principles for differentiating iPSC into retinal progeny have been laid down by previous work on mouse and human ESCs. However, the different propensity between iPSC and ESC for retinal differentiation brings into question the validity of the various protocols proposed. Most of these protocols rely on the initial spontaneous induction of retinal differentiation, but there is variability between cell lines. The lack of reproducibility by other laboratories also raises concern regarding their utility in the clinical setting. Nevertheless, there are two broad approaches: one by default differentiation of iPSC into neuroectodermal lineages (upon withdrawal of FGF2) and the other through directed differentiation by the addition of extrinsic molecules, such as growth and transcription factors.

A common approach in deriving retinal cells is to allow human iPSCs to overgrow as adherent layers. With the use of specific extracellular matrix in addition to certain inducing factors and proteins, iPSCs may be preferentially differentiated into RPE or photoreceptor phenotypes. For example, Tucker *et al.* described the formation of two-dimensional eyecup-like structures in a synthetic xeno-free culture substrate when skin keratinocyte-derived iPSCs were used [2]. After formation of small pigmented foci at around 45 days, these clumps expanded over 150 days. In some of these clumps, neural cells fill the centre, whilst in other colonies, pigmented cells wraps around in a C-shape around neural rosettes resembling a cross-section of an optic cup [2]. Similar two-dimensional eye-cup structures have also been reported by Jin *et al.* [53] and Reichman *et al.* [54].

**Table 1:** Characterisation of induced pluripotent stem cells, photoreceptor cells and retinal pigment epithelium.

Techniques of Characterisation	Induced Pluripotent Stem Cells	Photoreceptor Cells	Retinal Pigment Epithelium
Morphology (light microscopy)	Flat colonies; small and round cells; high nuclear to cytoplasmic ratio	Located in outer nuclear layer; cell bodies with processes; inner and outer segments	Monolayer; pigmentation; hexagonal
Morphology (electron microscopy)	N/A	Outer segment discs, myoid and ellipsoid segments, connecting cilia, basal body	Apical microvilli, basal infoldings, tight-junctional complexes, pigment granules
Cellular markers (pluripotency)	Surface: SSEA-3, TRA-1-60, TRA-1-81; Others: NANOG, SOX2, OCT4	Loss of OCT3/4, SOX2, NANOG	Loss of OCT3/4, SOX2, NANOG
Cellular markers (progenitors/precursors)	N/A	PAX6, CHX10, CRX, OTX2, NRL	PAX6, MITF
Cellular markers (differentiated/mature)	N/A	Phototransduction: recoverin, transducing, cGMP phosphodiesterase, retinal guanylate cyclase, cyclic-nucleotide gated channel, rhodopsin, cone opsins (S or L/M), arrestin; visual cycle	Visual cycle: RPE65, RLBP1, CRALBP; phagocytosis: FAK, MERTK; pigmentation: tyrosinase; growth factor: VEGF, PEDF, PDGF; membrane: Na/K ATPase, ZO-1, BEST1
Molecular	RT-PCR, bisulphite sequence analysis	RT-PCR	RT-PCR
Functional (*in vitro*)	Embryoid body formation	Patch recordings; response to white flash	Phagocytosis assay/rhodopsin clearance; fluid transport, polarised secretion of growth factors (PEGF/VEGF); transepithelial resistance
Functional (*in vivo*)	Teratoma assay in animal to identify all three germ layers	Cell transplantation to demonstrate rescue of visual function	Cell transplantation (RCS rat) to demonstrate rescue of visual function
Genetic	Karyotyping sequencing to look for new mutations	Sequencing to check no new mutations	Sequencing to check no new mutations

RT-PCR, Reverse transcription polymerase chain reaction; RCS, Royal College of Surgeons.

An alternative approach is to culture iPSCs as suspended aggregates to enable the formation of three-dimensional cellular structures. Recapitulation of ocular organogenesis through the formation of an optic cup structure using a serum-free suspension culture system was first demonstrated using murine ESC by Eiraku *et al.* and then human ESC by Nakano *et al.* [55,56]. More recently, Meyer *et al.* described human iPSC-derived cell aggregates with vesicle-like and non-vesicular configurations after 20 days of culture using successive media changes from embryoid body medium (four days) to neural induction medium (seven days) and, finally, to retinal differentiation medium [6,51]. The vesicle-like structures expressed CHX10, a marker of retinal progenitor cells, whereas the non-vesicular spheres expressed ISLET-1, a homeodomain protein involved in early forebrain development. Upon further differentiation, photoreceptor-like cells and RPE were derived from the vesicle-like structures. Similar optic vesicle-like structures have also been generated from lymphocyte-iPSC [4,36]. Zhong *et al.* recently reported three-dimensional laminated retinal cups generated from human iPSC with distinct populations of neural retinal cells interacting through synaptic junctions and photoreceptor cells capable of forming outer segment discs and responding to light [57]. However, it is important to note that efficiency in generating PAX6+ neuroectodermal cells amongst different iPSC clones can vary from 5% to 56% of the total cell population using the same protocol, highlighting the need for further investigation into the methods and screening criteria to identify the most suitable iPSC clone for retinal differentiation [51].

Irrespective of the protocol used for inducing retinal differentiation, the timing of the derivation of specific retinal cell types generally reflects the timeline of embryological development. This temporal recapitulation of embryogenesis by iPSC differentiation supports the notion that derivation of retinal cells is not directed, but rather the outcome of subcloning and culture in a permissive microenvironment. During the first month of embryonic development, the forebrain portion of the primitive anterior neuroepithelium gives rise to cells expressing markers specific for the eye field. Optic vesicles then develop from the eye fields at the end of the first month with cells expressing PAX6 and MITF. Retinal progenitors destined to become RPE preferentially express MITF, whereas those becoming neuronal cells downregulate MITF in response to increased CHX10 expression. Then, there is a 1–2 month(s) lag in the expression of *CRX* and opsin genes in neural retinal cells after the formation of RPE. Hence, the time to generate RPE from iPSC is typically around 4–6 weeks, whereas differentiation of photoreceptor precursors occurs at 2–3 months after retinal induction. Formation of outer segments and the development of light response was reported by Zhong *et al.* at six months after retinal induction [57]. Recently, Reichman *et al.* described a floating culture system for generating neuroretinal-RPE containing retinal progenitor cells within two weeks, which bypassed embryoid body formation and obviated the need for exogenous molecules, coating or Matrigel [54].

The ability to recapitulate retinogenesis using iPSC has tremendous potential for studying diseases that interfere with retinal development and non-cell autonomous mechanisms, in addition to those that cause post-natal cell autonomous retinal degeneration. On a practical level, there are still significant barriers to routine clinical use of this technology, since the generation of patient-specific retinal cells may take 4–6 months from the time of biopsy, and there is significant overhead infrastructure cost to maintain an iPSC laboratory. Future advancement in three-dimensional culture and differentiation techniques may one day enable iPSCs to differentiate into other structures of the eye, such as the choroid and sclera, thus expanding the use of iPSC in understanding complex retinal diseases, such as AMD and myopia.

#### 2.2.2. iPSC to Photoreceptor Cells

Hirami *et al.* described deriving photoreceptor cells using human iPSC from dermal fibroblast, serum-free embryoid body culture system, defined factors (Wnt and Nodal inhibitors) and subsequent plating of aggregates onto poly-d-lysine, laminin and fibronectin to generate retinal progenitors expressing *RX*, *PAX6* and *MITF* [58]. From Day 90, the application of retinoic acid and taurine to the culture system induced the expression of the photoreceptor marker, recoverin, in a quarter of the colonies by Day 120 (four months). Half of these recoverin-positive cells were also immune-positive for rhodopsin. Notably, only two of three iPSC lines could be differentiated into the retinal lineage, and functional assays of putative photoreceptor cells were not performed. Osakada *et al.* from the same group, at the RikenCenter for Developmental Biology, also reported a modified protocol using small molecules (casein kinase I inhibitor CKI-7, Rho-associated kinase inhibitor Y-27632 and ALK4 inhibitor SB-431542) to block Wnt and Nodal pathways to induce retinal progenitors [59]. This method has been used in generating photoreceptor cells from patients with *RP1*, *RP9*, *PRPH-2* and *RHO* mutations [7,53].

Meyer *et al.* [51] described a different culture system using embryonic stem cell medium without FGF2, then chemically-defined neural induction medium with N2 supplement followed by another chemically-defined retinal differentiation medium supplemented with B27. Rosettes were picked and selected for neurosphere culture and generation of optic vesicle-like structures. By Day 80, 14% of the neurospheres expressed rod- and cone-specific transcription factor Crx, within which 65% of the cells were expressing Crx. However, only 8% of the cells within Crx+ spheres expressed recoverin and/or opsin. This protocol was modified by Zhong *et al.* to generate photoreceptor cells (within laminated retinal cup structure) that express synaptic junction proteins, phototransduction molecules, to form outer segments and to respond to light stimulus [57]. The optic vesicle-like system was used to study the effect of *CHX10* mutation [4].

Several other groups have also described the derivation of photoreceptor cells from human iPSC (Table 2). There are many morphological, cellular, molecular, functional and genetic assays for the characterisation of iPSC-derived photoreceptors and their precursors, but there is no consensus on the minimum criteria (Table 1). Lamba *et al.* used their protocol for ESC [60] to derive photoreceptor cells from human iPSCs [52]. Although, they did not test the function of these cells, they demonstrated integration into mouse retina following sub-retinal transplantation. Mellough *et al.* combined the techniques described by Lamba *et al.* and Osakada *et al.* for deriving retinal cells from ESC and added activin A, Shh and T3 to enhance photoreceptor differentiation from human iPSCs [60,61,62]. Their three-step differentiation protocol involved inducing a neural lineage, then retinal progenitors and, finally, photoreceptor cells expressing blue, red and green opsin. For iPSC-derived photoreceptor cells to be used in human transplantation, animal-derived products should be avoided where possible. Tucker *et al.* and Sridhar *et al.* recently reported the generation of photoreceptor cells from iPSCs using a xeno-free system, where a synthetic culture surface (Synthemax cell culture surface) is used for iPSC derivation and retinal differentiation [37,63].

**Table 2:** Derivation of retinal photoreceptor (precursor) cells from human induced pluripotent stem cells.

Reference	Source of iPSC	Duration	Markers to Confirm Photoreceptor Lineage	Tests to Suggest Photoreceptor Cell Function	Transplant	Disease Modelling	Therapeutics Screening
Hirami *et al.* [58]	Human fibroblast	120 days	CRX, RCVRN, RHO	No	No	No	No
Osakada *et al.* [59]	Human fibroblast	120–140 days	CRX, PDC, PDE6b, PDE6c, RHO, GRK1, SAG, RCVRN	Molecules required for photo-transduction	No	No	No
Jin *et al.* [7]	Patient fibroblast	120 days	CRX, RCVRN, RHO, OPN1SW, OPN1LW	Patch clamp to detect voltage dependent channels 8-OHdG, caspase-3, acrolein, BiP, CHOP	No	Yes	Yes
Jin *et al.* [53]	Patient fibroblast *	120–150 days	CRX, RCVRN	BiP, CHOP	No	Yes	No
Meyer *et al.* [51]	Human fibroblast	80 days	CRX, RCVRN, Opsin	No	No	No	No
Meyer *et al.* [6]	Patient fibroblast	80 days	CRX, RCVRN	No	No	No	No
Phillips *et al.* [36]	Patient T-cells	108 days	CRX, RCVRN, S-OPSIN, RHO, CX36, SNAP-25, VGLUT1	Molecules required for synaptic function	No	No	No
Phillips *et al.* [4]	Patient T-cells	80 days	CRX, RCVRN, NRL, OPN1SW, PED6B	Molecules required for photo-transduction	No	Yes	No
Tucker *et al.* [3]	Patient fibroblast	33 days	RCVRN	No	No	Yes	No
Tucker *et al.* [2]	Patient keratinocyte	60 days	CRX, NRL, RCVRN, RHO, Acy Tubulin, OPN1SW, OPN1LW	GRP78, GRP94	Yes	Yes	No
Burnight *et al.* [64]	Patient fibroblast	90 days	CRX, RHO, OPN1SW, RCVRN, ROM1	No	No	No	Yes
Tucker *et al.* [37]	Patient fibroblast, Human keratinocyte and IPE *^,†^	90 days	CRX, NRL, RCVRN, RHO	No	No	No	No
Sridhar *et al.* [63]	Human fibroblast	60 days	CRX, RCVRN	No	No	No	No
Mellough *et al.* [62]	Human fibroblast	60 days	CRX, OPN1SW, OPN1LW, RHO, RCVRN, ARRESTIN 3	No	No	No	No
Reichman *et al.* [54]	Human fibroblast	49–112 days	CRX, NRL, RHO, R/G/B OPSIN, ARRESTIN 3, RECVRN	No	No	No	No
Zhong *et al.* [57]	Human fibroblast	175 days	CRX, OPN1SW, OPN1LW, RHO, PDE6α/β, Gtα, CNGA1/B1, RetGC1	Patch clamp-light induced response; outer segment disc formation on EM; molecules required for photo-transduction	No	No	No
Lambda *et al.* [52]	Human fibroblast	28 days	CRX, OTX2, NRL, RECVRN, AIPL-1, RHO, S-Opsin, Arrestin, PAX6, Blimp1	Molecules required for photo-transduction	No	No	No
Yoshida *et al.* [8]	Patient fibroblast	35 days	NRL promoter, recoverin	BiP, CHOP, BID, NOXA LC3, ATG5, ATG7	No	Yes	No

8-OHdG, 8-Hydroxy-2′-deoxygunosine (oxidative stress marker); BiP, Binding immunoglobulin protein; CHOP, C/BEP-homologous protein/DNA-damage-inducible transcript 3; RCVRN, Recoverin; * iPSC derived from integration-free iPSC; ^†^ iPSC derived from xeno-free culture.

#### 2.2.3. iPSC to Retinal Pigmented Epithelial Cells

**Figure 4:**
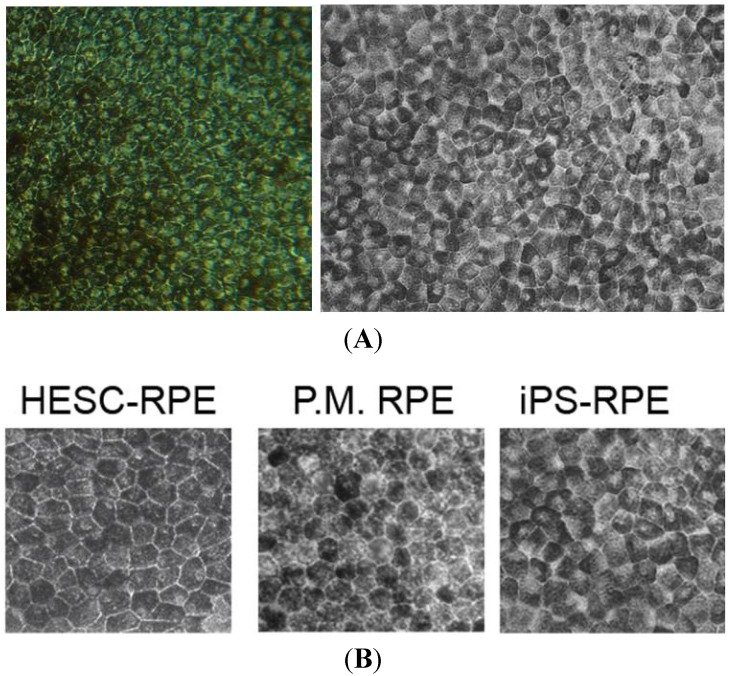
Morphology of the retinal pigment epithelium monolayer. (**A**) Hexagonal pigmented monolayer of retinal pigment epithelium derived from induced pluripotent stem cells; (**B**) Comparison of the morphology of retinal pigment epithelial stem cells derived from human embryonic stem cells (HESC), post-mortem (PM) eyes and induced pluripotent stem (iPS) cells.

Although the embryoid body culture system can generate RPE from iPSC, adherent culture has been favoured if RPE is the only cell that is required. Hirami *et al.* and Meyer *et al.* showed RPE differentiation occurs earlier than neural retinal progeny derivation [51,58]. Carr *et al.* and Buchholz *et al.* demonstrated that RPE differentiation from human iPSC can be achieved within four weeks, and these cells demonstrated morphological and molecular signatures of RPE, as well as *in vitro* and *in vivo* functional characteristics [65,66]. Morphologically, RPE derived from iPSCs is indistinguishable from RPE in post-mortem eye or human ESC-derived RPE (Figure 4).

Characterisation of iPSC-RPE involves morphological, cellular, molecular, functional and genetic assays (Table 1). Key morphological features include pigmentation, monolayer of hexagonal cells and electron microscopic features of apical microvilli, tight junctions, basal infoldings and cytoplasmic melanosomes. The molecular signature of RPE cells reflects their eye field origin (PAX6 and MITF) and function: RPE65 and CRALBP (retinoid cycle), MERTK (phagocytosis), bestrophin (modulating calcium flux in endoplasmic reticulum) and ZO-1 (tight junctions). *In vitro* functional assessment includes transepithelial resistance measurement, vascular endothelial growth factor (VEGF), platelet-derived growth factor (PDGF) and pigment epithelium-derived factor (PEDF) secretion, extracellular matrix production (laminin and type IV collagen) and phagocytosis assay using photoreceptor outer segments. *In vivo* functional assessment requires subretinal transplantation in an animal model of RPE or retinal dystrophy, such as the Royal College of Surgeons (RCS) rat, to assess the rescue of visual function [65,67,68,69]. Gene expression comparing human iPSC-RPE to adult and foetal RPE and other controls through microarray and hierarchical clustering analysis needs to be performed to verify similarity to target tissue [70].

For autologous transplantation of iPSC-RPE, immunogenicity, cell survival and tumourigenicity studies are also required. These have been addressed for iPSC-RPE specifically by Kamao *et al.* and Kanemura *et al.* [70,71] as part of a pre-clinical study in preparation for human iPSC-RPE autologous transplantation. The generation of patient-specific iPSC-RPE has also been performed in gyrate atrophy and Best disease (BD) [5,6]. The following section will illustrate clinical examples of the use of iPSC-derived retinal cells in genetic diagnosis, discovery of genotype/phenotype relationship, screening of pharmaco- and gene therapies and as a source of autologous cell therapy.

## 3. Clinical Use of Patient-Specific iPSC-Derived Retinal Cells

The ability to generate patient-specific retinal tissue and cells offers the opportunity to study the relationship between genetic variants and disease phenotypes. This technology is particularly useful in modelling IRDs, as there are around 200 genes with over 4200 known and many other unknown mutations causing disease phenotype in IRDs [72]. One in 2000–3000 individual are affected by IRDs, and these, collectively, are the most common cause of blindness in children and young adults. Given that emerging therapies for IRDs are likely to be mutation specific, it is important to identify pathogenic mutation(s) in every affected individual [73,74,75].

Many IRDs have a poor genotype-phenotype correlation; defects in a single gene may lead to a variety of disease phenotypes, while, on the other hand, a particular disease phenotype may be caused by mutations in a large number of different genes. Adding to the challenge of identifying causative mutations is the relatively common occurrence of X-linked and *de novo* autosomal dominant variants. Although Sanger sequencing of selected genes followed by targeted next-generation sequencing (NGS) can identify known pathogenic mutation in many individuals, exome or whole genome NGS combined with genetic linkage studies are required for the identification of novel mutations. Traditionally, these rare mutations (<1%) have been validated through functional modelling, mouse and zebrafish studies and replication of genotyping in large patient and relevant control cohorts. More recently, iPSC technology has also been used to confirm the pathogenicity of genetic variants and to unravel the molecular mechanism of disease phenotype through the *in vitro* study of cellular function and the histogenesis of iPSC-derived retinal tissue.

### 3.1. IPSC for Genetic Diagnosis and Modelling

#### 3.1.1. Confirming Pathogenicity of Mutation

Patient-specific iPSC has been used to confirm the pathogenicity of new rare genetic variants. For example, using NGS, single-strand conformation polymorphism screening and Sanger sequencing of a large validation cohort, Tucker *et al.* [3] identified a new mutation (Alu element insertion) in the male germ cell-associated kinase (*MAK*) gene causing rod-cone dystrophy. This was confirmed by examining and comparing the transcripts of *MAK* between iPSC and iPSC-derived photoreceptors from the patient and his unaffected sibling. The proband had no family history of retinal dystrophy, and the affected individual was heterozygous for pathogenic variants in *ABCA4* and *USH2A*. Using iPSC, they discovered a previously unrecognized exon 12 of the *MAK* gene that is expressed in cells differentiated into retinal precursors, but not in undifferentiated cells. This observation confirmed that the homozygous Alu element insertion in exon 9 is pathogenic by affecting the developmental switch from *MAK* bearing only exon 9 to a retina-specific transcript bearing both exons 9 and 12. The insertion of a 353-bp Alu repeat between codons 428 and 429 in exon 9 results in the insertion of 31 incorrect amino acids followed by a premature termination. In another study, Tucker *et al.* [2] reported the discovery of a new pathogenic variant of *USH2A* in another patient with rod-cone dystrophy who was presumed heterozygous for a pathogenic variant in *ABCA4* and *USH2A*. The second possibly disease-causing variant was found in intron 40 of *USH2A*, and this was confirmed by real-time PCR of patient-specific iPSC-derived photoreceptor precursor cells. A pseudoexon (IVS40) was formed by the intronic splice site mutation in the intervening sequence 40 of *USH2A*, and this caused a translation frameshift and a premature stop codon.

Lustremant *et al.* [76] examined the transcriptomics of human iPSC-derived neural stem cells and RPE from two patients with Leber congenital amaurosis (LCA). Although the pathogenic mutation was not known, they showed changes in the expression of 21 genes when compared to wild-type controls. Amongst these, three downregulated genes—*TRIM61*, *ZNF558* and *GSSTT1*—were related to the LCA disease process through protein degradation, altered transcription regulation and oxidation. With better understanding of the interactions between molecular pathways, detection of altered transcriptomics may help to narrow down candidate genes in this patient with LCA.

#### 3.1.2. Modelling Developmental Diseases of the Retina

The impact of known mutations on retinogenesis and cellular function has also been explored. The transcription factor CHX10 (*Caenorhabditis elegans* Ceh-10 homeo-domain-containing homolog 10), also known as VSX2 (visual system homeobox 2), has a critical role in the development of the retina. The very rare mutation in *VSX2* leads to severe malformation of the eye. Although animal models of this disease (the *VSX2*^−/−^ and (R200Q) *VSX2* mice) have contributed greatly to our understanding of the importance of *VSX2* in repressing MITF, production of the bipolar cell and maturation of the photoreceptors, it was not possible to confirm that the same mechanism occurs in humans. Phillips *et al.* [4] used iPSCs from a patient with a (R200Q) *VSX2* mutation to generate an embryoid body and then vesicles that recapitulated retinogenesis [6,36]. They confirmed previous observations in animal models and went a step further, using lentiviral *VSX2* overexpression to examine the reversibility of the developmental defect *in vitro*. Although suppression of MITF and enhanced photoreceptor maturation was achieved, bipolar cell markers were not restored by wild-type lenti-*VSX2*. Transcriptome analysis at Day 20 and 30 cells demonstrated overall upregulation of genes. Most of these were related to the WNT and TGFb signalling pathways that promote RPE differentiation. In contrast, the FGF pathway, which promotes neuroretinal differentiation, was downregulated.

The process of deriving retinal cells from iPSCs provides an opportunity for studying retinal development and developmental anomalies due to specific mutations that cause ocular and retinal dysgenesis (e.g., *MITF*, *PAX6*, *VSX2*, *CRB1*, *etc.*). However, terminally differentiated retinal cells from iPSCs can also be tested for altered cell function to understand degenerative diseases of the retina (see below). IRDs that have an earlier onset may be expected to demonstrate abnormality earlier in the differentiation protocol, whilst late onset IRDs (e.g., BD, pattern dystrophy, Sorsby fundus dystrophy and Doyne honeycomb retinal dystrophy) may not manifest altered cellular physiology unless the cells are aged and stressed *in vitro* to recapitulate senescence.

#### 3.1.3. Modelling Degenerative Diseases of the Retina

**Figure 5:**
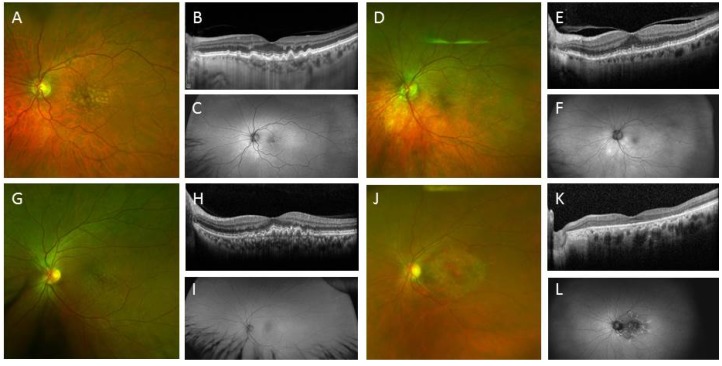
Clinical images of early age-related macular degeneration and its variants. (**A**) Colour photograph of the macula of a 72-year-old male showing soft drusen; (**B**) Optical coherence tomography (OCT) shows a sub-retinal pigment epithelial (RPE) deposit, which did not significantly alter fundus autofluorescence (**C**); (**D**) Colour photograph of the macula of a 78-year-old female showing reticular pseudo-drusen; (**E**) OCT shows deposits above the RPE, resulting in subtle hypo autofluorescent lesions (**F**); (**G**) Colour photograph of the macula of a 57-year-old female showing basal laminar drusen; (**H**) OCT shows a compact sub-RPE deposit forming a saw-tooth pattern, and these lesions were mildly hyper autofluorescent (**I**); (**J**) Colour photograph of the macula of an 83-year-old female showing dominant drusen or Doyne honeycomb retinal dystrophy; (**K**) OCT shows outer retinal layer loss; (**L**) The fovea was hypo autofluorescent due to RPE loss, and the linear radial drusen are seen as hyper autofluorescent streaks.

Both RPE and photoreceptor disease models have been created using patient-specific iPSCs. AMD (Figure 5) and two types of RPE dystrophies have been modelled: Best disease and gyrate atrophy.

Chang *et al.* reported reduced ZO-1 and RPE65 staining in iPSC-RPE generated from five atrophic AMD patients compared to two controls [38]. There was also increased accumulation of reactive oxygen species following exposure to H_2_O_2_ compared to controls. Furthermore, expressions of antioxidant genes (*HO-1*, *SOD2* and *GPX1*) were lower, whilst PDGF, VEGF and IGFBP-2 expressions were higher compared to controls. There were no details regarding the age of the two control subjects, and the AMD risk allele profiles for all seven subjects were not reported. Further studies are needed to replicate these findings by controlling other potential confounders. It is particular important that control subjects are age matched when iPSC is used to model late-onset degeneration as AMD. This is because AMD cannot be diagnosed until drusen is visualised, usually after the age of 50 years. Although the presence of risk alleles and family history increases the risk of developing AMD, these biomarkers are not 100% predictive. Therefore, somatic cells from young healthy individual cannot be used as controls, because it is not possible at this stage to predict if this subject will or will not develop AMD later in life.

**Figure 6:**
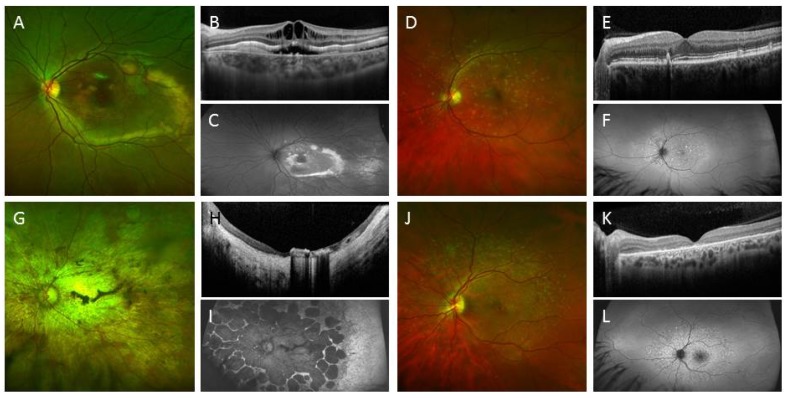
Clinical images of various types of inherited retinal diseases. (**A**) Colour photograph of the macula of a 10-year-old boy showing multifocal vitelliform lesions resulting from homozygous deletion of exon 2–6 of the *BEST1* gene; (**B**) Optical coherence tomography (OCT) shows intraretinal cystic change with sub-retinal fluid and vitelliform deposits; (**C**) Increased fundus autofluorescence was noted in the area of vitelliform deposits; (**D**) Colour photograph of the macula of a 57-year-old male showing yellow deposits due to pattern dystrophy of the retinal pigment epithelium (RPE); (**E**) OCT shows deposits above and below the RPE; (**F**) Multifocal hyper autofluorescent lesions are seen; (**G**) Colour photograph of the macula of a 56-year-old female showing extensive macular atrophy with cone-rod dystrophy due to two missense mutations in the *ABCA4* gene (c.2915 C > A and c.3041 T > G); (**H**) OCT shows severe retinal and choroidal atrophy with pigment migration into the fovea; (**I**) Extensive RPE loss resulting in wide-spread hypo autofluorescent lesions; (**J**) Colour photograph of the macula of a 32-year-old male showing retinal flecks with mild cone dysfunction due to two pathogenic mutations in the *ABCA4* gene (c.4139 C > T and c.6079 C > T); (**K**) OCT shows outer retinal layer loss to retinal atrophy; (**L**) Retinal flecks were hyper autofluorescent.

Heterozygous, compound heterozygous and homozygous mutation of the *BEST1* gene (bestrophin protein) can cause ocular disease characterised by abnormal RPE function, accumulation of debris between RPE and photoreceptors and a variable amount of retinal degeneration and ocular dysgenesis (Figure 6). There are over 100 mutations in *BEST1*, and the pathogenicity and molecular mechanism of RPE dysfunction arising from these mutations is not well understood. The traditional approach to study the effects of *BEST1* mutation is based on transfection of mutant *BEST1* gene into the human foetal RPE or Madin Darby canine kidney (MDCK II) epithelium cell lines. Sing *et al.* described the use of RPE derived from patient-specific iPSCs to study the impact of two mutations in the *BEST1* gene, (A146K and N296H) on RPE function. They demonstrated increased intracellular accumulation of autofluorescent materials compared to controls after long-term (3.5 months) feeding of the BD hiPSC-RPE with bovine photoreceptor outer segments (POS, 50/cell) and decreased net fluid transport. Conflicting data at 4 and 24 hours were shown regarding delayed degradation of POS when overfed with FITC-POS (50 *vs.* 20 POS per cell). As expected, there was no significant difference in the RPE differentiation potential of BD hiPSC compared to controls. BD hiPSC-RPE also had a similar transepithelial resistance, level of *BEST1* mRNA and localisation pattern of the mutant bestrophin compared to controls. The dysfunction in mutant bestrophin was found to be associated with altered endoplasmic reticulum (ER)-mediated calcium homeostasis. Furthermore, there was increased expression of genes involved in regulating oxidative stress (*GPX1*, *SOD2*) and iron homeostasis (*TRF*, *TRFR*) after long-term (3.5 months) POS feeding [5]. In this study, the genetic background between the cell lines was not controlled by genomic editing of the *BEST1* mutation into the control iPSC or out of the BD hiPSC (see below). Nevertheless, this is a good example where early onset disease with a well-characterised clinical disease phenotype can be recapitulated *in vitro*. It is not known if similar cellular abnormalities can also be detected in iPSC-RPE from patients with the much commoner late-onset vitelliform macular dystrophy due to other types of *BEST1* mutations. Phagocytosis assay may be a better readout for iPSC-RPE generated from patients with a known genetic defect that impairs phagocytosis, such as *MERTK* or *REP-1* mutation (choroideremia).

In a very different RPE dystrophy, gyrate atrophy, homozygous mutation in the ornithine-δ-aminotransferase gene (*OAT*) leads to RPE damage and loss, leading to severe peripheral and central vision loss. RPE has been successfully generated from iPSCs derived from the fibroblasts of a patient with *OAT* mutation (A226V). Enzyme activity of OAT within the iPSC-RPE can be measured [6]. Correction of the *OAT* mutation by bacterial artificial chromosome-mediated homologous recombination restored the enzymatic activity [77]. There are other RPE dystrophies resulting from mutations affecting visual cycle enzymes or regulators (e.g., the acyltransferase, LRAT, the isomerohydrolase, RPE65, the dehydrogenase, RDH12, and RPGR and RLBP1). Deriving iPSC-RPE from patients with various genetic mutations in these enzymes or regulators may also provide opportunities to understand genotype-phenotype molecular mechanisms and variability.

Many genes are involved in photoreceptor cell degeneration. Clinically, there are two broad classes of disease phenotypes based on electrophysiology: macular dystrophy, which is limited to the macular region, and retinal dystrophy, where the entire population of photoreceptors (central and peripheral) is affected. Generalised retinal dystrophy can affect cones or rods predominantly. Different mutations in one gene, such as *ABCA4*, can have varied disease phenotypes, including macular dystrophy, cone dystrophy, cone-rod dystrophy or rod-cone dystrophy (Figure 6). The overlap between various disease phenotypes and causative mutation reinforces the importance to understand the molecular mechanisms of genotype-phenotype relationships.

Amongst the genes causing rod-cone dystrophy, the molecular effect of mutations in *RHO*, *RP1*, *RP9*, *PRPH-2* and *USH2A* have been studied using iPSCs. Jin *et al.* [7] used iPSCs from patients with *RHO*, *RP1*, *RP9* and *PRPH-2* to generate rod photoreceptor precursors. They demonstrated reduced rod cell number at Day 120 in iPSC-rod precursors derived from patients with *RP9* mutation (early-onset retinal degeneration). This effect was seen in *RHO*, *RP1* and *RHO* mutation at Day 150, whereas no rods were detectable for the iPSC line carrying the *RP9* mutation. They also showed that the *RP9* mutant iPSC-rod precursors had increased oxidative stress. In contrast, the *RHO* mutant iPSC-rod precursors had mislocalisation of the rhodopsin protein and increased expression of ER stress markers, which might be explained by the accumulation of unfolded rhodopsin [7,53]. Tucker *et al.* also examined the impact of *USH2A* mutation on photoreceptor precursor cells. They described increased expression of *GRP78* and *GRP94* in iPSC-derived photoreceptor precursor cells, indicative of ER stress related to protein misfolding. It is important to note that controls used in these reports were not genetically matched, *i.e.*, the mutation was not removed by genomic editing.

A major limitation of these genotype-phenotype studies is the choice of controls. For metabolic syndromes or disease with early manifestation (ocular dysgenesis or early degeneration), healthy related or unrelated controls will be adequate, because of the robust and rapid cellular manifestation of the phenotype. However, controls used for studying diseases with delayed onset will need to be genetically matched to avoid the cofounding effect of the (1) genetic background, (2) retinal cell differentiation process and (3) genetic alteration introduced during the process of iPSC reprograming. Yoshida *et al.* confirmed the effect of *RHO* mutation (E18K) on rod precursor cell ER stress responses, apoptosis markers and autophagy activation by repairing and introducing the mutation in the affected and control (provided by Yamanka laboratory) cell lines, respectively, using a helper-dependent adenoviral vector gene transfer [8]. Similar genetic control was also reported for gyrate atrophy-iPSC-RPE, where restoration of the *OAT* gene in the iPSC using bacterial artificial chromosome-mediated homologous recombination resulted in normalisation of *OAT* enzyme activity in iPSC-RPE [6,77].

Once the clinically-relevant cellular phenotype and readouts can be defined for the specific genetic variant and mutation, high throughput analysis will need to be developed to enable a large number of therapeutics to be screened across the potential thousands of genetic variant cell lines from patients with IRDs. Although this is not yet possible, there are several examples where this has been reported on a smaller scale (see below). Further investigations are needed to determine if late-onset retinal or macular degeneration, such as reticular pseudodrusen (Figure 5), basal laminar drusen (Figure 5), pattern dystrophy of the RPE, vitelliform macular dystrophy and mutations arising from *EFEMP-1* (Doyne honeycomb retinal dystrophy, Figure 5), *TIMP-3* (Sorsby fundus dystrophy) or *CTRP5* (late-onset retinal degeneration), can also be modelled through derivation of retinal cells from iPSCs. The readout or functional assay for each of these diseases may differ significantly because of variable environmental contribution to the disease phenotype and diverse molecular pathogenic mechanisms. For example: Sorsby fundus dystrophy may be caused by deposition of abnormal extracellular protein (TIMP-3); Doyne honeycomb retinal dystrophy may be related to activation of unfolded protein response due to misfolded fibulin-3 (EFEMP-1); and late-onset retina degeneration may be associated with abnormal intracellular protein aggregates, as well as extracellular deposition [78,79,80,81]. There are also many IRDs that affect retinal cells downstream from the photoreceptors, such as congenital stationery night blindness and X-linked retinoschisis [82]. Patient-specific iPSC-derived laminated retinal structures may be ideal for the study of pathophysiology of these inner retinal IRDs, since many of the genes involved in these diseases (e.g., *NYX*, *CACNA1F*, *GRM6*, *TRPM1*, *CABP4*, *CACNA2D4* and *RS1*) are involved in the extracellular matrix of neural retina and the synaptic interaction between photoreceptor and bipolar cells [83].

### 3.2. IPSC for Therapeutics Development and Treatment

#### 3.2.1. IPSC for Drug Screening

Current therapeutic modalities in IRDs are aiming to preserve residual cells or replace missing cells. Because gene therapies and many pharmacotherapies will need to be tailored for individual genetic variants or mutations, iPSCs provide an ideal platform for pre-clinical therapeutic and toxicology testing. There are several examples in both AMD and IRDs, where pharmaco- and gene therapies are tested using iPSC.

Chang *et al.* tested the protective effect of curcumin on iPSC-RPE derived from AMD patients [38]. They showed that curcumin had a beneficial effect on H_2_O_2_-induced cell death and reactive oxygen specifies generation in both control and AMD iPSC-RPE. Exposure of curcumin also increased the expression of *HO1*, *SOD2* and *GPX1* and decreased the expression of *PDGF*, *VEGF* and *IGFBP-2* in AMD iPSC-RPE. Whether this also occurred in control iPSC-RPE was not reported.

*OAT* deficiency in the RPE leads to a buildup of ornithine and a reduction in high-energy creatine phosphate. In some patients, the *OAT* enzymatic activity can be reversed by a high dose pyridoxine (vitamin B6) supplement, because of the effect of *OAT* mutation on the binding affinity to pyridoxine. Clinically, vitamin B6 responsiveness is tested in patient fibroblasts. Although based on the fibroblast assay, A226V *OAT* mutation is not responsive to vitamin B6, Meyer *et al.* has demonstrated that iPSC-RPE from this patient is, in fact, responsive to vitamin B6 based on an *in vitro* dose titration experiment. Therefore, this patient has directly benefited from iPSC disease modelling and pharmacotherapy screening.

Jin *et al.* [7] demonstrated the benefit of α-tocopherol (vitamin E) on improving the survival of iPSC-rod precursor cells in the *RP9* mutation. They also confirmed no toxic effect from 1.6 μM of ascorbic acid (vitamin C) and β-carotene (vitamin A) on iPSC-rod precursors affected by *RP1*, *RP9*, *PRPH-2* and *RHO* mutations. In addition to vitamins, modulators of signal pathways have also been screened using iPSC. Yoshida *et al.* showed that inhibition of mTOR (using rapamycin or PP242), activation of AMP kinase (using AICAR), inhibition of apoptosis signal-regulating kinase (using NQDI-1) and inhibition of protein synthesis (using salubrinal to inhibit eukaryotic translation initiation factor 2 subunit α phosphatase) can reverse the increased ER stress and apoptosis and autophagy marker expression seen in *RHO* mutant iPSC-rod precursor cells [8].

#### 3.2.2. IPSC for Testing Gene Therapy

In addition to screening prospective pharmacological agents, iPSC has also been used to test the efficacy of gene therapy approaches in LCA and choroideremia, which aim to deliver the *CEP290* and *REP-1* genes, respectively, to retinal cells. CEP290 is a centrosomal protein involved in ciliogenesis and ciliary trafficking. Mutation in CEP290 leads to abnormality of the inner and outer segments of cone cells, resulting in early-onset severe visual loss. Previous ocular gene therapy used adeno-associated viral (AAV) vectors for delivery of the *RPE65* gene. However, the large size of CEP290 precludes the use of AAV, and therefore, lentivirus is necessary for gene delivery. Burnight *et al.* [64] described the method to package full-length human CEP290 into a lentiviral vector and demonstrated restoration of a ciliogenesis defect in LCA patient-derived fibroblasts. Although they also demonstrated expression of wild-type CEP290 after lentiviral transduction of the iPSC-photoreceptor precursor cells, they did not examine the impact of this on cone development and the formation of inner or outer segments. In contrast, Vasireddy *et al.* [84] showed successful transfection of AAV2 carrying full-length human *REP-1* cDNA into iPSCs rather than transfection into differentiated retinal cells, the presumed target cell primarily affected in choroideremia. They used a prenylation assay to confirm restoration of *REP-1* function following AAV2. *REP-1* infection of the iPSC. There was also improved trafficking of RAB27 in iPSCs, because of prenylation by viral-derived REP-1. The efficiency and toxicity of iPSC transduction was compared to patients’ fibroblasts, but not patient-derived iPSC-RPE. These two examples demonstrate the potential of iPSC in pre-clinical studies of patient-specific gene therapy.

#### 3.2.3. iPSC for Cellular Therapy

Cell therapy for retinal disease aims to replace (1) photoreceptors and/or (2) supporting cells that provide trophic and metabolic support to prevent further degeneration of remaining photoreceptors. The main challenges in establishing clinically acceptable cell therapy for retinal disease are: patient selection, surgical technique, carrier system and choice of cell source. Each of these questions has its complexity in several dimensions. However, the use of iPSCs as a source for retinal cell transplantation is one of the most exciting, but also complex and challenging, issues facing scientists, clinicians, pharmaceutical companies and regulatory authorities.

In comparison to human ESC, the use of iPSC-derived cells for cell therapy has the additional requirement of quality control associated with the surgical procedure in harvesting patient somatic cells, isolation of a single cell type from the tissue biopsy, reprogramming vectors and techniques, methods of genomic editing in the case of IRDs and techniques of hiPSC clone selection and storage. Some of these steps have been defined in standard operating procedures for the production of clinical-grade iPSCs from retrovirus reprogramming. However, this is yet to be established for the numerous published non-integrating reprogramming methods.

Derivation of clinical-grade retinal cells from hESC has been conducted in GMP facilities, and it is currently being used in phase I/II clinical trials as hESC-RPE suspension for Stargardt disease, geographic atrophy due to AMD and myopic atrophic macular degeneration (Clinical Trial: NCT01469832, NCT01345006, NCT01344993, NCT02122159) and hESC-RPE patch graft for wet AMD (NCT01691261). There is only one human trial using iPSC-RPE, at the RIKEN Center for Developmental Biology (CDB), Japan. In the CDB trial, a monolayer of iPSC-RPE without substrate is used to treat neovascular AMD after a course of ranibizumab injections. One patient, in her 70s with wet AMD, has already received her own iPSC-RPE as a 1.3 × 3.0-mm cell patch at Kobe City Medical Center General Hospital in September [85]. This group has published data to support the *in vitro* and *in vivo* function of the iPSC-RPE in performing the visual cycle [68]. They also demonstrated suppression of the tumour-forming potential of iPSC by iPSC-RPE following subcutaneous transplantation in NOD/Shi-*scid*/IL-2Rγ^null^ (NOG) mice. They postulated that pigment epithelium-derived factor secreted from iPSC-RPE or RPE of host tissue can cause apoptotic cell death of iPSC [86]. Following from this, the tumourigenicity of iPSC-RPE was also tested in nude, severe combined immune deficiency (SCID), non-obese diabetic (NOD)-SCID and NOG mice in the subretinal and subcutaneous location. They observed no tumour formation at 6–12 months following transplant [71]. Immune reaction to autologous iPSCs has not been studied, but Kamao *et al.* [70] demonstrated a lack of immune response after one year when nonhuman primate iPSC-RPE was transplanted as an autograft into the subretinal space. This study also confirmed no evidence of tumour formation following monkey iPSC-RPE autograft [70]. The RIKEN CDB has already enrolled patients with neovascular AMD for iPSC-RPE transplantation after the disease is stabilised with anti-VEGF therapy. Although the functionality of the graft seems to be well characterised, significant work still needs to be done in developing the optimal surgical instrumentation, technique and approach in resurfacing the RPE in the submacular space and patient selection for optimal visual and anatomical outcome. It is not known if iPSC-RPE survive, as multiple small patches of epithelial monolayer are superior to cell suspension and not inferior to a single large sheet of epithelial-substrate complex that can cover the entire macular region. The importance of restoring damaged Bruch’s membrane during iPSC-RPE replacement therapy in AMD cannot be underestimated, as this is considered as an important aspect of the pathophysiology of AMD [87].

*In vitro* genomic editing or mutation repair of harvested somatic cells, iPSCs or iPSC-derived retinal cells will provide patients with IRDs the opportunity to receive autologous cell therapy. For some IRDs that develop late-onset degeneration, genetic mutation correction in the patient-specific iPSCs may not be necessary [2]. This is relevant in the situation where the strategy is to transplant (1) iPSC-derived retinal supporting cells that are not affected by the mutation (e.g., RPE cells for ABCA4 retinopathy); or (2) iPSC-derived retinal cells affected by the mutation, but it has delayed non-cell autonomous effects due to reduced trophic factor release (e.g., rod precursor cells for the cone preservation function through the release of rod-derived cone viability factor) [88]. For replacement of cone photoreceptor cells in cone dystrophy or RPE in RPE dystrophy, *in vitro* genomic editing may be required to allow iPSCs to differentiate into mature photoreceptors or the RPE phenotype and to ensure long-term survival of the autograft. Examples of *in vitro* gene therapy testing have been described in the previous section. Future strategies may include site-specific transcription activator-like effector nucleases (TALEN) or clustered regularly interspaced short palindromic repeats (CRISPR) based genome editing techniques, where the mutation is edited through a double-strand break (DSB) and off-target mutagenesis minimised by single-guide RNA. Following induction and subsequent homology-directed repair (HDR), the corrected gene will remain under the normal endogenous expression control elements.

## 4. Conclusions

Since the description of iPSCs in 2006, there has been an exponential increase in the translation of this technology towards understanding disease mechanisms and the discovery of therapeutics. The full potential is yet to be realised because of the complexity and variations in reprograming technology and retinal differentiation protocols. The relationship between the clinical disease phenotype and the molecular and cellular features of specific genetic variants in iPSC-derived retinal cells is still poorly understood. These issues will need to be resolved for iPSC-derived retinal tissue to become clinically relevant and useful in modelling retinal dysgenesis and degeneration. Standardisation and development of high throughput technology to interrogate specific retinal progeny derived from iPSCs will facilitate screening of genetic mutation and testing of pharmacologic and gene therapy in rare IRDs. Early data from several publications show that this may have a direct benefit to the patient [2,3,64,84]. iPSCs as a source of autologous cells are an attractive option, but there is a significant barrier to overcome for this to become scalable to treat large numbers of patients. However, progress in cell culture automation and refinements of reprogramming methods will undoubtedly facilitate the translation of iPSC-derived tissue into clinically applicable personalized cell therapy. Despite the mountain of challenge, the escalating costs of biologic therapy for treating neovascular and atrophic AMD and the suffering from irreversible childhood blindness due to IRDs, further ophthalmic translational research in iPSCs is worthy of the costly investment.
